# Development of a Bacterial Lysate from Antibiotic-Resistant Pathogens Causing Hospital Infections

**DOI:** 10.3390/microorganisms13081831

**Published:** 2025-08-06

**Authors:** Sandugash Anuarbekova, Azamat Sadykov, Dilnaz Amangeldinova, Marzhan Kanafina, Darya Sharova, Gulzhan Alzhanova, Rimma Nurgaliyeva, Ardak Jumagaziyeva, Indira Tynybayeva, Aikumys Zhumakaeva, Aralbek Rsaliyev, Yergali Abduraimov, Yerkanat N. Kanafin

**Affiliations:** 1 Scientific and Analytical Center “Biomedpreparat” LLP, Stepnogorsk 021500, Kazakhstan; 2 Scientific Center for Anti-Infectious Drugs, Almaty 050000, Kazakhstan; 3 National Holding “QazBioPharm”, Astana 010000, Kazakhstan; a.rsaliyev@qbp-holding.kz (A.R.);; 4 Faculty of Natural Sciences, L.N. Gumilyov Eurasian National University, Astana 010000, Kazakhstan

**Keywords:** bacterial lysate, antibiotic resistance, respiratory pathogens, clinical isolates, French press homogenization

## Abstract

Biotechnological research increasingly focuses on developing new drugs to counter the rise of antibiotic-resistant strains in hospitals. This study aimed to create bacterial lysates from antibiotic-resistant pathogens isolated from patients and medical instruments across hospital departments. Identification was performed based on morphological, cultural, and biochemical characteristics, as well as 16S rRNA gene sequencing using the BLAST algorithm. Strain viability was assessed using the Miles and Misra method, while sensitivity to eight antibacterial drug groups and biosafety between cultures were evaluated using agar diffusion. From 15 clinical sources, 25 pure isolates were obtained, and their phenotypic and genotypic properties were studied. Carbohydrate fermentation testing confirmed that the isolates belonged to the genera *Escherichia*, *Citrobacter*, *Klebsiella*, *Acinetobacter*, *Pseudomonas*, *Staphylococcus*, *Haemophilus*, and *Streptococcus*. The cultures exhibited good viability (10^9^–10^10^ CFU/mL) and compatibility with each other. Based on prevalence and clinical significance, three predominant hospital pathogens (*Klebsiella pneumoniae* 12 BL, *Pseudomonas aeruginosa* 3 BL, and *Acinetobacter baumannii* 24 BL) were selected to develop a bacterial lysate consortium. Lysates were prepared with physical disruption using a French press homogenizer. The resulting product holds industrial value and may stimulate the immune system to combat respiratory pathogens prevalent in Kazakhstan’s healthcare settings.

## 1. Introduction

The problem of antibiotic resistance is of global importance, as this issue exists in all countries and poses a significant global health threat. The problem equally affects both highly developed industrial countries and developing nations, regardless of a country’s economic status [1,2,3,4]. Antibiotic resistance, a type of antimicrobial resistance, refers to bacteria’s ability to withstand treatment with antibacterial drugs. Antimicrobial resistance more broadly includes resistance in bacteria, fungi, and parasites to modern treatment methods [3,5]. Antibiotic resistance has become a pressing issue in the 21st century. In most regions of the world, including Kazakhstan, multidrug-resistant strains have become widespread [6].

The most serious life-threatening infections are caused by a group of resistant microorganisms, which have been referred to by WHO and the Infectious Diseases Society of America (IDSA) as the ESKAPE pathogens, since they effectively “escape” the effects of antibacterial drugs. This acronym stands for the main antibiotic-resistant pathogens: *Enterococcus* spp., *Staphylococcus aureus*, *Klebsiella pneumoniae*, *Acinetobacter baumannii*, *Pseudomonas aeruginosa*, and *Escherichia coli* [7,8,9,10]. They are assigned the highest “priority status” due to the significant threat they pose to human health [11]. However, the list of these bacteria continues to expand.

Antimicrobial resistance develops through the evolution of bacteria. This phenomenon is natural in the lifecycle of microorganisms; however, several factors contribute to its intensification. Resistance to antimicrobial agents develops in microbes, not in humans or animals. Key contributing factors include inappropriate antibiotic use, the presence of antimicrobial agents in food products (used to treat or prevent animal disease, promote growth, or suppress microbial growth, particularly in dairy), and the use of antibiotics in cosmetics. Self-medication, often based on previous experiences, internet resources, and advice from pharmacists, is another contributing factor [12]. Hospital environments are traditionally viewed as sources of antibiotic-resistant microorganisms, with infections acquired in these settings termed hospital-acquired or nosocomial infections. These infections affect 5–10% of patients [13]. The issue of antibiotic-resistant strains is further complicated by the reduced production of new drugs. Monoresistant organisms are becoming multidrug resistant and, eventually, pan-resistant [14,15].

External factors are also key contributors to this problem. The migration of people, birds, and animals facilitates the spread of bacteria from one region to another. Large-scale food trade also promotes the global spread of antibiotic-resistant microbes. Sources contributing to antibiotic resistance include industrial, pharmaceutical, and hospital waste; wastewater; and agricultural runoff [1,16,17]. Evidence of migration is provided by a study showing the epidemic spread of *P. aeruginosa* ST235 VIM-2 clone across Russia, Belarus, and Kazakhstan. The majority of isolates were sensitive only to polymyxins [18]. Thus, antimicrobial resistance poses a significant threat to public health.

A practical solution is the use of microbial cell structures, specifically bacterial lysates (BLs), which inhibit their target microorganisms [19,20,21,22]. BLs are products of bacterial cell breakdown (lysis), containing fragments of bacterial cell walls and intracellular contents. They contain short-chain peptides, free amino acids, oligo- and monosaccharides (e.g., glucose, galactose), volatile fatty acids, and vitamins. Lysates possess immunomodulatory, anti-inflammatory, anti-allergic, antioxidant, and membrane-stabilizing properties. They preserve the natural microflora, do not impair gastrointestinal function, and support immunity development against specific pathogens [23]. During the lysis process, bacteria lose their viability but retain several important properties [24,25]. Bacterial preparations were associated with the first successes in immunotherapy (biotherapy) of human diseases. At the turn of the 19th and 20th centuries, New York physician William B. Coley successfully used injections of a mixture of killed *Str. pyogenes* and *Serr. marcescens* in patients with malignant tumors, known as “Coley’s toxins” or “Coley’s vaccines” [24].

Locally acting lysates form an even layer on the mucosal surface, enhancing absorption. BLs of local action increase the amount of secretory immunoglobulin sIgA, which protects the mucous membranes and prevents microorganisms from “settling” on them. Systemic drugs trigger an immune response by activating T- and B-lymphocytes and macrophages. The mechanism of action of immunostimulatory drugs involves interaction with the mucosal lymphoid tissue, which is constantly exposed to pathogenic antigens. It includes the lymphoid tissues of the intestines, bronchi, nose, lacrimal glands, mammary glands, salivary glands, and urogenital tract. This leads to the production of IgA antibodies, which, in their secretory form (sIgA), perform a protective function by covering and agglutinating microbial cells, exerting bacteriostatic action, preventing endothelial adhesion, and neutralizing toxins [24,25].

Several BL-based drugs are known, including ismigen [26], lantigen B [27], and OM-85 [28,29], and are used to treat respiratory infections. Urostim, urovac, and others are used for treating various urogenital tract infections [29]. All of them are made based on lysates from bacterial pathogens such as *S. aureus*, *Str. pyogenes*, *Str. viridans*, *Str. pneumoniae*, *Kl. pneumoniae*, *Kl. ozaenae*, *H. influenzae*, *E. coli*, and others. Suárez N et al. noted that OM-85 and ismigen, representing chemical and mechanical lysis, respectively, are the most studied BLs [28]. It has been established that ismigen is effective in preventing upper respiratory tract infections among adults and children, which is reflected in a reduction in the number of recurrences [23,26,30]. It consists of *S. aureus*, *Str. pyogenes*, *Str. viridans*, *Str. pneumoniae*, *Kl. pneumoniae*, *Kl. ozaenae*, *H. influenzae*, and *N. catarrhalis*. One of the modern and highly effective bacterial immunomodulators is OM-85 (Broncho-Munal and Broncho-Vaksom), which contains lyophilized lysates of the most common pathogens of acute respiratory diseases, such as *S. pneumoniae*, *Kl. pneumoniae*, *St. aureus*, *S. pyogenes*, *Bran. catarrhalis*, *H. influenzae*, *Kl. ozaenae*, and *S. viridans* [30,31]. The crucial role of BLs in modern medicine is reflected in the works of Mario Di Gioacchino and others [24,25,32].

The development of bacterial lysates represents a promising approach to counteracting antibiotic-resistant infections by enhancing host immune defenses without contributing to further resistance. Based on the urgent need for alternative therapeutic strategies and the growing threat of multidrug-resistant pathogens, the objective of this study is to develop bacterial lysates derived from antibiotic-resistant clinical isolates. This work aims to contribute to the expanding field of immunomodulatory therapies, offering potential new tools for preventing and managing infections caused by resistant microorganisms.

## 2. Materials and Methods

### 2.1. Study Objects

The study involved 25 clinical bacterial isolates, including 10 strains from the *Enterobacteriaceae* family (*Escherichia* spp.—5, *Citrobacter* spp.—2, *Klebsiella* spp.—3) and isolates from the genera *Pseudomonas* (6 strains), *Staphylococcus* (6 strains), *Streptococcus* (1 strain), *Acinetobacter* (1 strain), and *Haemophilus* (1 strain) (Table 1). The microorganisms were obtained from various biological materials of patients, including wound exudates, mucosal surfaces, bodily secretions, and the contents of medical tubing. The biological materials were provided by the “Scientific Center for Anti-Infectious Drugs” (Almaty, Kazakhstan). These cultures were selected based on their inclusion in the recommended list for hospital antibiotic resistance surveillance and with viability exceeding 10^6^ CFU/mL, meeting the standards for industrial and reference strains.

Isolation of a pure culture usually involves three stages: obtaining the enrichment culture, isolating the pure culture, and determining its purity [25,33,34,35]. For bacterial isolation, the following media were used: meat peptone broth (MPB), meat peptone agar (MPA), blood agar, egg yolk-salt agar, Mueller–Hinton agar and broth, MacConkey agar, Baird-Parker agar, chocolate agar, and Endo medium. All media were purchased from HiMedia Laboratories Pvt. Ltd., Mumbai, India. Cultivation was carried out at 37 °C for 18–24 h.

Purity was assessed by the homogeneity of growth on the medium, the uniformity of cells in the smear, and their taxonomic characteristics. Subsequent studies examined cultural properties, morphological features observed using Gram-stained smear microscopy, and biochemical characteristics according to *Bergey’s Manual of Determinative Bacteriology* [33]. Based on these characteristics, the bacterial phenotype was studied, and taxonomic affiliation was established. Upon confirmation of culture purity and characteristic consistency, the isolated colonies grown on solid media were subcultured for storage on slanted agar and cryopreserved in MPB at −20 °C with 20% (*v*/*v*) glycerol added [34].

### 2.2. Determining Microbial Viability

To evaluate the maximum viability of microbial cultures, the Miles and Misra serial dilution method was applied, ensuring accurate quantification of viable cells [36].

The initial cultures used for microbial viability testing were grown for 18–24 h on slanted agar media. After incubation, bacterial cells were washed off the agar surface using sterile physiological saline and transferred into sterile tubes. The inoculum was prepared according to the McFarland turbidity standard (10 units), corresponding to approximately 9 × 10^8^ CFU/mL. The turbidity of the bacterial suspension was visually compared to the McFarland standard and adjusted if necessary by adding saline or culture suspension. This ensured the desired concentration was reached before proceeding with serial dilution and viability testing.

A working suspension was prepared, and titration was performed up to the 10^10^ dilution. A new pipette was used for each dilution as failure to follow this rule could lead to inaccurate results. A sterile pipette tip was used to place a 20 µL drop of bacterial suspension onto each sector of the Petri dish. Then, the inoculated plates were incubated, and the number of grown colonies was determined. The number of cells in 1 mL of the studied substrate was calculated using Equation (1):

(1) $$M=a\times 10^{n}\times V\times 50$$

where

M—number of cells in 1 mL;

a—average number of colonies from the plated dilution;

n—dilution factor;

V—volume plated (mL);

50—conversion factor from µL to mL.

All experiments were performed in triplicate. The average values and standard deviation were calculated.

### 2.3. Determination of Antibacterial Drug Resistance

The sensitivity of the isolated cultures to antibiotics was assessed using the standard disk diffusion method [37]. The procedure involved the following steps: (1) preparation of nutrient agar medium; (2) preparation and standardization of microbial suspensions to a turbidity equivalent to a McFarland standard of 10, followed by inoculation of the agar surface; (3) application of antibiotic disks; (4) incubation under appropriate conditions; and (5) measurement and interpretation of inhibition zones to determine susceptibility. Antibiotic disks were obtained from two sources: the Scientific Research Center of Pharmacotherapy (SRCPh, Saint Petersburg, Russia) and HiMedia Laboratories Pvt. Ltd. (Mumbai, India). The following antibiotic disks and concentrations were used:

- SRCPh (Russia): imipenem (10 µg), meropenem (10 µg), gentamicin (10 µg), benzylpenicillin (1 unit), erythromycin (15 µg), amoxicillin (20 µg), streptomycin (10 µg), tetracycline (30 µg), chloramphenicol (levomycetin, 30 µg), and ampicillin (10 µg).
- HiMedia (India): cefepime (30 µg), ceftriaxone (10 µg), levofloxacin (5 µg), and pefloxacin (5 µg).

A broad spectrum of antibiotics was used to allow evaluation of resistance across both Gram-positive and Gram-negative bacterial strains. For quality control, *E. coli* 209-P and *S. aureus* 209-P were used as reference strains. These strains were obtained from the culture collection of the N.F. Gamaleya Research Institute of Epidemiology and Microbiology (Moscow, Russia) and were also used to assess nutrient media sterility and performance.

### 2.4. In Vitro Biocompatibility Determination

Biocompatibility was assessed in vitro by evaluating the co-growth or mutual inhibition of drug-resistant strains. The exponential culture of the investigated strain was streaked onto the surface of an agar medium in a Petri dish, and other cultures were subsequently inoculated perpendicularly. Incubation was carried out at 37 °C for 18–24 h [38].

### 2.5. Identification of Bacterial Isolates Using 16S rRNA Gene Sequencing

DNA was extracted from each uncharacterized isolate using the “DNA/RNA-C-FACTOR” kit, following the manufacturer’s instructions. DNA concentration was determined spectrophotometrically using the NanoDrop 1000 device (Waltham, MA, USA).

#### 2.5.1. Amplification of the 16S rRNA Gene Fragment

PCR was performed using universal primers [39] 8f (5′-AgAgTTTgATCCTggCTCAg-3′) and 806R (5′-ggACTACCAgggTATCTAAT-3’) in a total volume of 30 µL. The PCR mix contained 5 ng of DNA, 1 U of Maxima Hot Start Taq DNA Polymerase (Fermentas, Vilnius, Lithuania), 0.2 mM of each dNTP, 1× PCR buffer (Fermentas), 2.5 mM of MgCl_2_, and 10 pmol of each primer. The PCR amplification program included initial denaturation at 95 °C for 3 min, followed by 32 cycles of 95 °C for 30 s, 55 °C for 40 s, and 72 °C for 60 s. A final elongation step was performed at 72 °C for 10 min. The PCR was carried out using a GeneAmp PCR System 9700 (Applied Biosystems, Carlsbad, CA, USA).

#### 2.5.2. Electrophoretic Analysis of Amplification Products

Amplified target DNA fragments were analyzed using 1.5% agarose gel electrophoresis with ethidium bromide as the intercalating agent for visualization. Electrophoresis was performed in a horizontal electrophoresis chamber (PowerPac, Hercules, CA, USA) with a BioRad Electrophoretic Bath (Hercules, CA, USA) as the current source. In addition, 1× TAE buffer was used as the electrode buffer.

#### 2.5.3. Nucleotide Sequencing and Analysis

The PCR products were cleaned of unbound primers and dNTPs using magnetic bead-based purification. DNA purification using magnetic silica-coated beads was performed as previously described [35,40]. The sequencing reaction was carried out using the BigDye^®^ Terminator v3.1 Cycle Sequencing Kit (Applied Biosystems) according to the manufacturer’s instructions, followed by fragment separation on an automated genetic analyzer, the 3730xl DNA Analyzer (Applied Biosystems). The nucleotide sequences were analyzed and assembled into a consensus sequence using the SeqMan software (version 6.1, DNAStar). Afterward, terminal fragments (primer sequences and low-quality regions) were removed, and identification was performed using the BLAST algorithm in GenBank (https://blast.ncbi.nlm.nih.gov/Blast.cgi, accessed on 13 March 2025).

### 2.6. Bacterial Lysate Preparation

Initially, the purity of the working culture was assessed using Gram staining and microscopy. The working cultures were grown in test tubes containing 10 mL of MPA for 24 h at 37 °C. Then, 10 mL of bacterial suspension was added to 1 L flasks containing 500 mL of MPA for biomass propagation, and cultivation was conducted at 37 °C for 48 h. After this, the suspension was transferred to test tubes and diluted with sterile distilled water in a 1:1 ratio. Next, the test tubes were placed in a freezer at −80 °C for 24 h. Afterward, the test tubes were removed from the freezer and thawed completely at room temperature. The suspension was then transferred to sterile syringes for further processing. The syringes were thoroughly washed with 90% ethanol and sterilized using UV light. Finally, the BLs were obtained through a physical method using a cell disintegrator (French press homogenizer, NanoGenizer, Rohs, Shanghai, China) in a sterile environment.

### 2.7. Cell Disintegrator Operation

For the operation of the cell disintegrator, 96% ethyl alcohol and sterile distilled water are required. All procedures were carried out under sterile conditions. After loading the program, the mode for washing the internal pistons of the apparatus was set to a power range of 60 to 80.

Power settings were as follows:

- 60—moderate power, suitable for delicate cell processing, minimizing damage to the biomaterial;
- 70—medium, balanced for effective cell disintegration with minimal risk of component breakdown;
- 80—high, which accelerates the destruction of cell walls but may increase the risk of damaging more sensitive structures. The pressure was not to exceed 12.9 kPa.

The pistons were first washed with ethyl alcohol, followed by sterile distilled water at the same power settings. The pressure during washing was not to exceed 15.3 kPa at power levels 60–80. This treatment was performed after each sample, as well as before and after each session [41]. A syringe containing biomass was placed into the piston marked “Input”, and a sterile syringe was inserted into the opposite piston. In the “System Settings” section, Cycle 2 and Mode 100 were selected, with a maximum pressure of 30,000 kPa, depending on the material’s consistency. The process was then initiated. After the biomass was processed and transferred to the second syringe, it was removed and reloaded into the first piston for repeated processing. The biomass syringe was processed a total of three times. The apparatus was sterilized after each cycle. The pressure typically fluctuated between 25.5 and 27.8 kPa. After completing operation with the homogenizer, the internal piston components were sterilized, and the program was shut down using the “System Settings” menu.

Microbial viability in the resulting substance was assessed using the Miles and Misra serial dilution method with up to four dilutions in MPA-containing Petri dishes. After incubation, if no growth was observed in any dilution, the biomass cultures were frozen and prepared for lyophilization. For this purpose, the Heto Power Dry PL6000 lyophilizer (Costa Mesa, CA, USA) was used. To initiate lyophilization, the chamber temperature was reduced to −80 to ensure effective moisture removal. Once the required temperature was reached, the frozen biomaterial was loaded into the lyophilizer. The samples were pre-frozen to −60 to −70 °C for 24 h to ensure optimal moisture removal during drying. Lyophilization was performed over 24–48 h to obtain a stable, dried substance with preserved quality.

## 3. Results

### 3.1. Isolation of Microbial Strains and Determination of the Maximum Viability Index

In this study, the research objects were extended-spectrum β-lactamase (ESBL)-producing strains of *Enterobacteriaceae*, *Acinetobacter*, *Pseudomonas*, *Staphylococcus*, *Haemophilus*, and *Streptococcus*. The rationale for selecting these was their relevance as nosocomial pathogens in Kazakhstan, where bacteria from the genera *Pseudomonas* spp., *Acinetobacter* spp., *Enterococcus* spp., *Haem. influenzae*, streptococci, staphylococci, and enterobacteria are commonly encountered [7,42,43,44]. These same microorganisms also fall under the category of antibiotic-resistant strains recognized globally [7,42,43,44]. Thus, our research aimed to obtain BLs from antibiotic-resistant strains isolated from patient biotopes.

All cultures were pure and demonstrated abundant, uniform growth on Petri dishes and in test tubes containing slanted agar media. The purity of the cultures was confirmed by microscopic examination, which showed that the cells were morphologically homogeneous and matched taxonomic characteristics. The coccal forms included *Streptococcus* and *Staphylococcus*, while the rod-shaped forms included *E. coli*, *Klebsiella*, *Citrobacter*, *Pseudomonas*, *Acinetobacter*, and *Haemophilus*. This study confirmed that the 25 microbial cultures corresponded to their respective species and genera. The next stage of the work involved investigating the maximum colony-forming units (CFU), as this is a key indicator for both collection and industrial microbial strains. A culture must be viable and exhibit a cell viability greater than 10^6^ CFU/mL. The number of viable cells was assessed using the sectoral plating method following serial dilutions. Mean values and standard errors are presented in Table 1.

Three strains exhibited a concentration of 10^10^ CFU/mL, while the majority (22 strains) demonstrated values of 10^9^ CFU/mL. The CFU/mL values were logarithmically transformed, resulting in log CFU/mL values ranging from 9.0 to 11.48. Thus, the 25 cultures from various genera were confirmed to be pure and viable. The cultures were preserved using cryopreservation methods (glycerol and sucrose) for future use [34,45] to prevent their loss. The samples were stored on slanted agar media at 4 °C. Cultures must be preserved using multiple methods to avoid strain loss or deterioration of their phenotypic characteristics. Maintaining strains in active form and preserving their valuable traits are essential prerequisites for virtually all types of microbiological work, from initial study to application in the production of various biopreparations.

**Figure 1:**
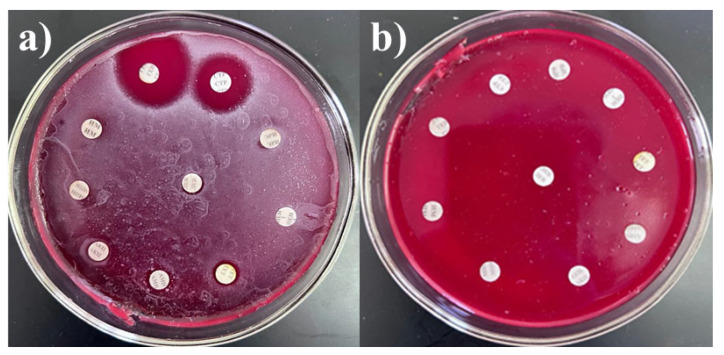
Antibiotic resistance of (**a**) *S. haemolyticus* 4 BL and (**b**) *Str. pneumoniae* 5 BL strains.

To select drug-resistant pathogens, the cultures were tested for resistance to antibacterial agents, including carbapenems, fluoroquinolones, and cephalosporins. To assess multidrug resistance (MDR), additional tests were conducted against other groups of antibiotics commonly used in clinical practice, such as macrolides, aminoglycosides, penicillins, tetracyclines, and chloramphenicols. Although some antibiotics are not effective against certain bacteria, all drugs were tested to assess the resistance profiles of the cultures. The interpretation of antibiotic resistance was based on the diameter of the inhibition zones. Resistance was defined as the absence of an inhibition zone or the presence of only a minimal inhibition zone around the antibiotic disk, indicating that the microorganism is not susceptible to the antibiotic tested. The results were presented in Table 2 and Figure 1, with *S. haemolyticus* 4 BL and *Str. pneumoniae* 5 BL shown as examples. *S. haemolyticus* 4 BL demonstrated susceptibility to gentamicin and streptomycin, while exhibiting resistance to all other tested antibiotics.

Each strain exhibited a distinct antibiotic resistance profile. *E. coli* isolates were resistant to 10 of the 14 antibiotics tested, including amoxicillin, ampicillin, benzylpenicillin, tetracycline, erythromycin, chloramphenicol, cefepime, ceftriaxone, levofloxacin, and pefloxacin. These antibiotics belong to several major classes, including penicillins, tetracyclines, macrolides, chloramphenicols, fluoroquinolones, and cephalosporins, indicating severely limited treatment options for *E. coli* infections.

The two *Citrobacter* spp. strains demonstrated resistance to amoxicillin, ampicillin, benzylpenicillin, erythromycin, and cefepime. *Klebsiella* strains were resistant to carbapenems, penicillins, erythromycin, tetracycline, ceftriaxone, and pefloxacin.

*Staphylococcus* spp. isolates showed broad resistance to meropenem, imipenem, amoxicillin, ampicillin, benzylpenicillin, erythromycin, lincomycin, tetracycline, ceftriaxone, and levofloxacin. These antibiotics represent the carbapenem, penicillin, tetracycline, macrolide, fluoroquinolone, and cephalosporin classes.

The *Str. pneumoniae* strain 5 BL was resistant to all antibiotics tested, indicating a highly multidrug-resistant phenotype.

*P. aeruginosa* strains exhibited resistance to multiple antibiotic groups, including penicillins, tetracyclines, macrolides, chloramphenicols, aminoglycosides, fluoroquinolones, and cephalosporins. The *Acinetobacter* strain 24 BL was resistant to penicillins, gentamicin, streptomycin, and cephalosporins. *Haem. influenzae* displayed resistance to imipenem, gentamicin, streptomycin, tetracycline, and cephalosporins.

**Figure 2:**
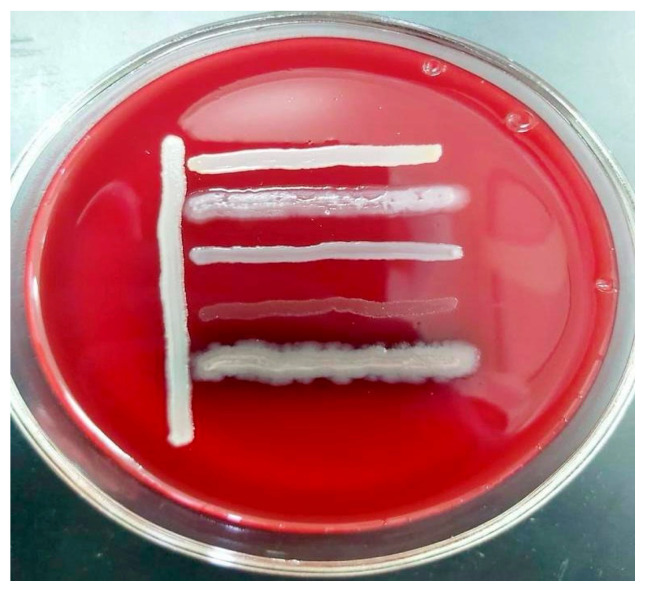
In vitro compatibility assessment of *Acinetobacter* spp. 24 BL with *Str. pneumoniae* 5 BL, *S. epidermidis* 7 BL, *P. aeruginosa* 13 BL, *P. aeruginosa* 14 BL, and *P. aeruginosa* 15 BL on blood agar.

In summary, the bacterial isolates demonstrated high levels of multidrug resistance, posing a serious challenge for clinical treatment. Resistance was observed across nearly all major antibiotic classes, including carbapenems, fluoroquinolones, cephalosporins, macrolides, aminoglycosides, penicillins, tetracyclines, and chloramphenicols. The majority of the strains were compatible with one another, with the exception of *Str. pneumoniae* 5 BL and *S. epidermidis* 7 BL, which demonstrated incompatibility with all *Pseudomonas* strains (Figure 2).

This compatibility information was taken into account during the development and formulation of bacterial lysates. The microbial cultures studied in this work were therefore evaluated as valuable for industrial applications.

### 3.2. Development of Bacterial Lysates Based on Drug-Resistant Pathogens

Lysate production was performed by freezing the bacterial cultures at −80 °C, followed by thawing and mechanical disruption using the cell disintegrator. The disintegrator was operated in either single-pass or cyclic mode.

Each strain required individual optimization due to differences in culture density and incubation times, which ranged from 24 to 48 h. The single-pass mode proved insufficient, as approximately 60% of the cells were destroyed, as confirmed by microscopy (Gram-stained smears) and by growth observed on nutrient agar plates. Consequently, a cyclic mode was adopted, with optimization of processing cycles, operating pressure, suspension concentration, and additional parameters, including force, power, and temperature. Different numbers of cycles (one, two, and three) were tested to achieve optimal disintegration results (Table 3). The evaluation was based on the reduction of viable cell count in serial dilutions. The initial concentration of cells was 9 × 10^8^ CFU/mL, prepared according to a McFarland standard of 10 IU.

An assessment of the Spearman correlation between the 1st and 3rd cycles was conducted. The Spearman correlation coefficient (ρ) was 1.000, indicating a perfect positive (direct) relationship. According to the Chaddock scale, the strength of this relationship was classified as functional. The number of degrees of freedom (df) was 23. The critical value of the Spearman correlation coefficient for this df was 0.398. Since ρ_obs_ > ρ_crit_, the correlation was statistically significant (*p* < 0.05), indicating that the relationship was not random and the dependence between the features was strongly associated. The first culture samples were subjected to one cycle of treatment. The first culture samples underwent one treatment cycle. The next day, growth was observed in 17 cultures at dilutions ranging from 10^2^ to 10^8^ CFU/mL on Petri dishes. The second samples were subjected to two cycles of treatment, resulting in CFU values at dilutions of 10^2^–10^5^ CFU/mL. No growth was observed above 10^6^ CFU/mL in any culture. The third samples were treated with three cycles, and growth was observed at dilutions of 10^2^–10^3^ CFU/mL. A total of 20 cultures showed a concentration of 10^2^ CFU/mL. Thus, the number of treatment cycles was not the primary factor in cell disruption. However, a three-cycle treatment was selected based on its ability to achieve the required CFU reduction.

*Varying Suspension Concentrations of Cultures.* To determine the most effective formulation, experiments were conducted using suspensions of varying concentrations. When using the French press, denser biomass suspensions resulted in significantly higher pressure and flow values, on average 5 to 7 kPa greater, compared to more dilute suspensions. However, dense biomass frequently clogged the press piston, significantly delaying the processing time. Therefore, a medium-density suspension was selected, as it maintained appropriate working pressure while minimizing clogging.

*Pressure.* The maximum allowable pressure was determined experimentally. A value of 30,000 kPa was used in this study. As the apparatus does not allow manual pressure adjustment, only the maximum allowable pressure could be set, as per the manufacturer’s instructions. Although the device permits up to 35,000 kPa, the limit was set to 30,000 kPa for this study.

*Power.* Power settings ranged from 10% to 100%. For the experiments, 100% power was applied in combination with three treatment cycles. After each treatment, the apparatus was sterilized with ethyl alcohol and sterile distilled water.

*Temperature of Culture Suspensions.* Proteins in bacterial cultures denature at temperatures above 50 °C. To prevent this and improve process efficiency, a cooling system was developed for use during cell disruption. Cryogens were used, and during the sterilization of the internal parts of the French press, the syringe with the biomass was placed between the cryogens and cooled during this time. Additionally, prior to processing, the prepared biomass was stored in a low-temperature refrigerator for 24 h to enhance cell disruption.

Fifteen different modes, varying in power and number of cycles, were tested (Table 4).

**Figure 3:**
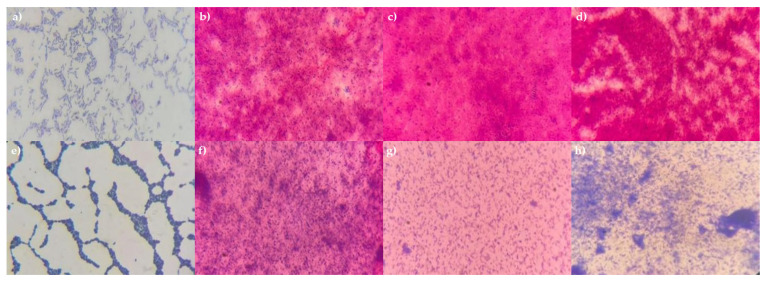
Microphotographs of *P. aeruginosa* 13 BL and *S. haemolyticus* 4 BL cells before and after treatment. Gram staining, magnification ×1000. (**a**) *P. aeruginosa* 13 BL (before treatment); (**b**) *P. aeruginosa* 13 BL, 1 cycle, 100% power; (**c**) *P. aeruginosa* 13 BL, 2 cycles, 100% power; (**d**) *P. aeruginosa* 13 BL, 3 cycles, 100% power; (**e**) *S. haemolyticus* 4 BL (before treatment); (**f**) *S. haemolyticus* 4 BL, 1 cycle, 100% power; (**g**) *S. haemolyticus* 4 BL, 2 cycles, 100% power; (**h**) *S. haemolyticus* 4 BL, 3 cycles, 100% power.

The various mode options did not result in complete cell destruction. Mode 15, which involved three cycles at 100 percent power, was the only setting that proved effective for all tested bacteria. This was demonstrated using *P. aeruginosa* 13 BL and *S. haemolyticus* 4 BL, in which complete cell destruction was confirmed by plating on solid nutrient media (no growth observed) and by microscopy (Figure 3). Thus, the optimal conditions for obtaining bacterial cell lysates were identified.

**Figure 4:**
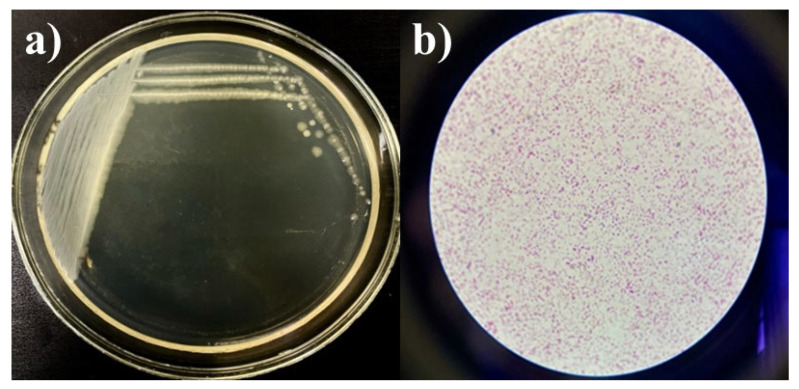
Growth of the consortium on MPA (**a**) and Gram-stained smear (**b**), magnification ×100.

Strains *Kl. pneumoniae* 12 BL, *P. aeruginosa* 3 BL, and *Acinetobacter* sp. 24 BL were selected based on a concentration of 10^9^ CFU/mL, resistance to multiple antibiotics (up to eight, representing all major drug groups), and efficient growth characteristics. The selection was also supported by bacteriological studies in Kazakhstan, which identified these strains as key contributors to hospital-acquired complications, particularly respiratory tract infections [21,44,46,47]. To create the consortium, the cultures were grown on slanted agar media, washed, and then cultured in test tubes containing MPB and slanted MPA at 37 °C for 24 h. To verify the final composition of the consortium, growth was assessed on a nutrient medium using the depletion streak method. The presence of microorganisms was confirmed microscopically, and the maximum CFU/mL of the consortium was determined using the Miles and Misra method. The cultures were streaked onto Petri dishes using the depletion streak method, and robust growth was observed (Figure 4a). Gram-stained smears revealed a mixed association of the three strains (Figure 4b). No contamination was detected. The maximum concentration of the consortium was 10^9^ CFU/mL.

**Figure 5:**
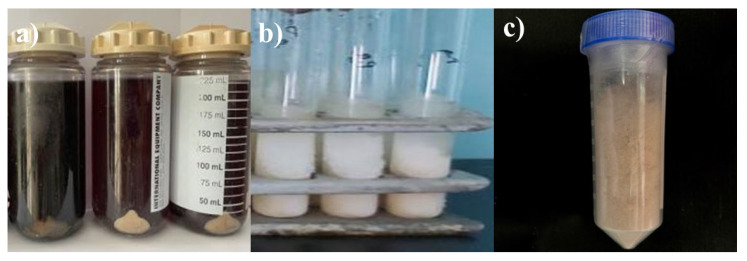
Samples from the *Kl. pneumoniae* 12 BL, *P. aeruginosa* 3 BL, and *Acinetobacter* sp. 24 BL consortium at different stages of processing: (**a**) biomass before treatment (live cells), (**b**) after disruption using a French press, and (**c**) lyophilized lysate.

To increase biomass yield, the consortium was initially grown in test tubes with MPB, followed by cultivation on MPA plates. The resulting biomass was washed and transferred into flasks containing 1 L of MPB, which significantly increased production. The consortium was then homogenized at 100 percent power for three cycles and subsequently dried using lyophilization. The results are shown in Figure 5. The yield was 9 to 10 g of dry mass per liter of culture liquid for each strain. These were then combined in equal proportions (1:1:1) to form the final consortium.

Thus, the lysate, consisting of three inactivated antibiotic-resistant strains (*Kl. pneumoniae* 12 BL, *P. aeruginosa* 3 BL, and *Acinetobacter* sp. 24 BL), processed using a protocol of three cycles at 100% power, was confirmed to be pure and free of contamination. The cells were completely destroyed, as confirmed by plating on solid nutrient media (no growth observed) and by microscopy, which showed no intact cells.

### 3.3. Identification of the Consortium Cultures

Genotyping was performed on these three cultures, as it was necessary to confirm their genus and species affiliation. Additionally, a genetic passport was required for depositing the strains in culture collections.

The issue of diseases exacerbated by the antibiotic resistance of infectious agents is multifactorial and requires the involvement of beneficial live microorganisms for human health. The challenge is that many pathogens resist a broad spectrum of antibacterial drugs, which complicates treatment and often renders it prolonged or ineffective. One of the proposed solutions is the development of preparations based on bacterial lysates. The cultures included in the consortium were identified using nucleotide sequence analysis of the 16S rRNA gene. This method enables accurate determination of the genus, species, and strain-level affiliation of microorganisms. Identification and differentiation of microbial species represent a critical stage in microbiological research, as they provide essential insights into the biological properties and potential functional roles of the isolates. This is carried out based on the study of a complex of phenotypic and genotypic traits that allow for comparison of the gene sequences and phenotypic characteristics of the investigated cultures with museum strains from national or international collections. Bacterial identification was carried out using direct nucleotide sequencing of the 16S rRNA gene fragment, followed by determining the nucleotide identity with sequences deposited in the International Gene Bank database, as well as constructing phylogenetic trees with nucleotide sequences of reference strains. DNA extraction was performed, followed by determining its concentration using a spectrophotometer. The main wavelengths used to measure DNA absorption are 260 nm (UV region) and 280 nm (protein component of DNA). The DNA concentration for the 12 BL was 122.6 ng/μL, for the 3 BL was 247.3 ng/μL, and for the 24 BL was 206.8 ng/μL.

**Figure 6:**
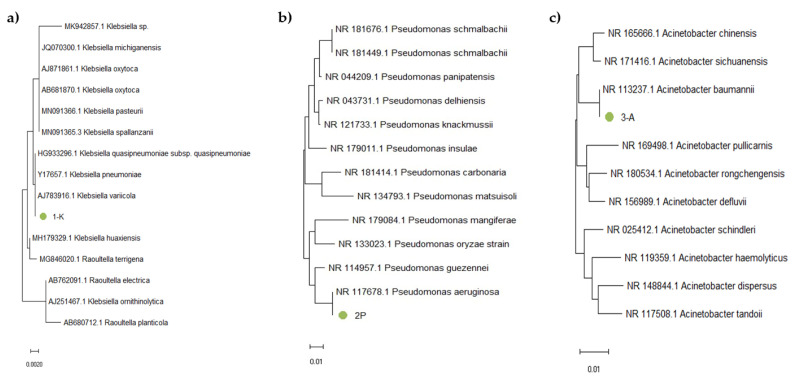
Phylogenetic tree based on 16S rRNA gene fragment analysis of samples (**a**) 1-K, (**b**) 2-P, and (**c**) 3-A.

The obtained nucleotide sequences were analyzed and merged into a single sequence using SeqMan software (DNA Star), with the removal of low-quality fragments and primer sequences. The obtained sequences were identified in GenBank using the BLAST algorithm. Based on the availability of nucleotide sequences in international databases such as GenBank, RDP-II, and Bacterio.net, we also constructed phylogenetic trees using 16S rRNA gene sequences of reference strains. The analysis included 16S rRNA gene nucleotide sequences of phylogenetically related microorganisms. For phylogenetic tree construction, we used the Mega X software (version 10.2.6). The Muscle algorithm was used to align the nucleotide sequences, and the trees were constructed using the Neighbor-Joining (NJ) method. The results are shown in Figure 6.

**Figure 7:**
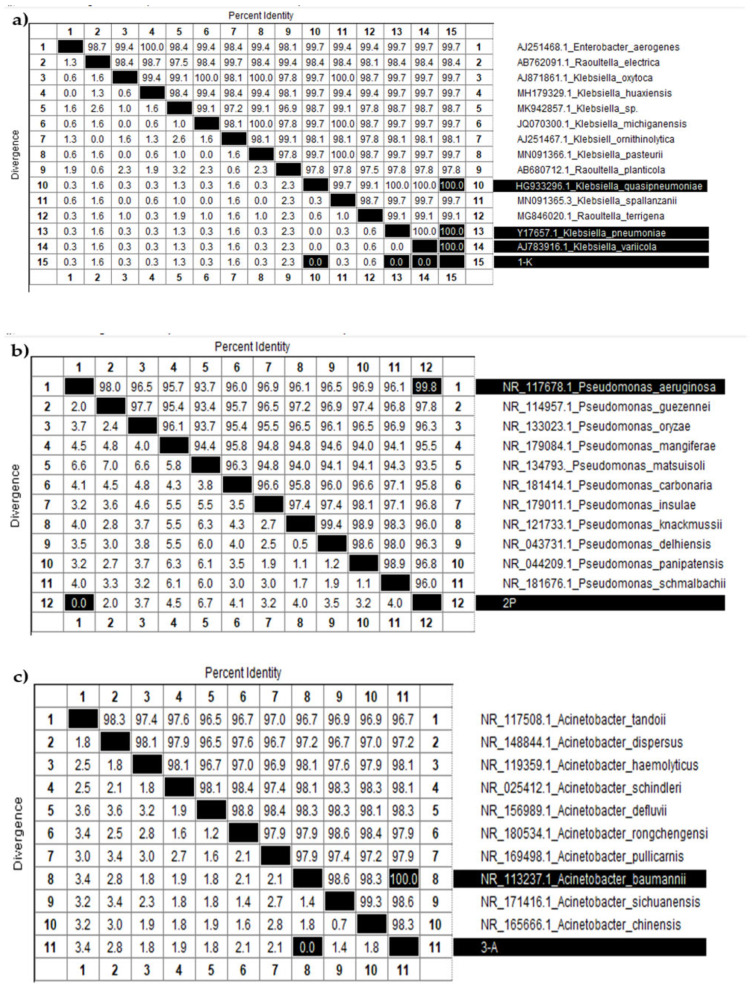
Identity and divergence analysis of nucleotide sequences among various (**a**) *Klebsiella*, (**b**) *Pseudomonas*, and (**c**) *Acinetobacter* species.

Sample 1-K (Figure 6a) clustered within the same branch as *Kl. pneumoniae*, *Kl. quasipneumoniae*, and *Kl. variicola*. Due to the high sequence similarity of the 16S rRNA gene among these closely related subspecies, precise identification requires additional analysis, such as sequencing of protein-coding genes or performing a detailed phenotypic characterization. Sample 2-P (Figure 6b) was located in the same branch as *P. aeruginosa*, which confirmed the phenotypic data indicating that this strain is *P. aeruginosa*. Sample 3-A (Figure 6c) clustered within the same branch as *A. baumannii*. For precise identification, further analysis was performed by examining the nucleotide sequences of protein-coding genes as well as conducting phenotypic characterization (Figure 7).

Figure 7a displays a table summarizing the percent identity and divergence of nucleotide sequences among various *Klebsiella* species. The diagonal, highlighted in black, indicates 100 percent identity for each sample compared to itself. The target sample “1-K” (sample 15, shown at the bottom of the table) demonstrated complete identity and zero divergence with three other samples: 10 (*K. quasipneumoniae*), 13 (*K. pneumoniae*), and 14 (*K. variicola*). These results confirm that sample 1-K belongs to the *Klebsiella* genus and is genetically most similar to these species. However, due to the high similarity of 16S rRNA sequences among closely related subspecies, further molecular or phenotypic analyses are needed to determine its exact species.

Figure 7b presents a similar analysis for *Pseudomonas* species. Sample “2-P” (sample 12) showed the highest similarity to sample 1 (*P. aeruginosa*), with 99.8 percent identity and no divergence. Likewise, Figure 7c shows data for *Acinetobacter* species, where sample “3-A” (sample 11) exhibited complete identity with sample 8 (*A. baumannii*), strongly supporting its classification as *A. baumannii*.

These identification results, based on 16S rRNA gene fragment analysis, confirmed that sample 1-K belongs to the *Klebsiella* genus (closely related to *K. pneumoniae*, *K. quasipneumoniae*, and *K. variicola*), sample 2-P corresponds to *P. aeruginosa*, and sample 3-A to *A. baumannii*. These assignments were further validated by phylogenetic trees constructed using the Neighbor-Joining method, providing strong molecular confirmation of species-level identity.

Since the Klebsiella 12 BL culture exhibited features common to three closely related species, biochemical testing was performed for phenotypic confirmation (Table 5).

Thus, the biochemical analysis performed for the *Klebsiella* 12 BL culture confirmed its classification as *Kl. pneumoniae*. Therefore, the identification results confirmed the genus and species names of the strains: *Kl. pneumoniae* 12 BL, *P. aeruginosa* 3 BL, and *A. baumannii* 24 BL.

## 4. Discussion

The challenge lies in the fact that many pathogens exhibit resistance to a broad spectrum of antibacterial drugs, which complicates treatment and often renders it prolonged or ineffective. One proposed solution is to develop drugs based on lysates of antibiotic-resistant pathogens such as *E. coli*, *Proteus* spp., *Klebsiella* spp., *Acinetobacter* spp., *P. aeruginosa*, *S. aureus*, and *Str. pyogenes*, among others. These taxonomic groups are selected for the production of pharmacologically active modulators because they are major causative agents of both infectious and non-infectious diseases, including wound and burn infections. Furthermore, they exhibit resistance to a broad spectrum of antibacterial agents, which is among the most clinically concerning features of these bacteria.

The global prevalence of multidrug-resistant strains, which are responsible for hospital outbreaks, continues to increase at an exponential rate. Studies conducted in Kazakhstan also reflect a broad spectrum of polyresistant pathogens. For example, a microbiological investigation was carried out to assess the microbial landscape and antibiotic susceptibility of strains isolated from adult patients admitted to intensive care and resuscitation units between 2010 and 2014 [48]. Of the 928 clinical samples, 781 strains were isolated. Pathogens were recovered from the respiratory and urinary tracts, as well as from wounds and drainage systems. The highest proportion of isolates came from respiratory tract samples (45.3%, 354 isolates), followed by urinary tract samples (28.5%, 223 isolates), and wound swabs (13.0%, 102 isolates). Among them, 52.3% (409 strains) were Gram-negative bacteria. Non-fermenting Gram-negative organisms predominated, accounting for 34.4% (269 isolates), including *A. baumannii* (20.9%, 164 isolates) and *P. aeruginosa* (13.4%, 105 isolates). Other isolates belonged to the Enterobacteriaceae family, including *Enterobacter* spp., *E. coli*, and *Kl. pneumoniae*, which demonstrated high resistance to third-generation cephalosporins and carbapenems. Gram-positive bacteria included *Enterococcus* spp., coagulase-negative staphylococci, and *S. aureus* (1.5%). Similar results were observed in studies of children in the cardiac intensive care unit [49].

The following study [21] presents data on the prevalence and molecular epidemiology of metallo-β-lactamase (MBL)-producing Gram-negative bacteria in Russia (1998–2010), Belarus, and Kazakhstan (2005–2010). Analysis of nosocomial strains isolated during several multicenter epidemiological studies in Russia revealed a rapid increase in the proportion of MBL-positive *Pseudomonas aeruginosa* isolates from 4.5% in 2002–2004 to 20.3% in 2006–2007. These findings suggest that the spread of MBL-producing *P. aeruginosa* strains in neighboring countries has reached a critical supranational level. The associated resistance to nearly all antibiotic classes, except polymyxins, significantly limits treatment options for infections caused by these strains. In patients with urinary tract diseases in Russia, Belarus, and Kazakhstan, a total of 1260 microorganisms were isolated, primarily enterobacteria. The most common species were *E. coli* and *Kl. pneumoniae*, both of which exhibited resistance to the main groups of antibiotics [47].

In the pediatric cardiac surgery department of Astana, Kazakhstan, a previous study conducted between 2010 and 2019 was continued [46]. The results of the study for 2020–2022 show that the most common causative agents of respiratory tract infections were *S. aureus*, *Kl. pneumonia*, *A. baumannii*, *P. aeruginosa*, *E. coli*, and MDR *Candida* spp. [50]. Another study [51] aimed to determine the frequency and resistance level of *A. baumannii* in infectious lesions in children during the period 2018–2021. The percentage of occurrence increased annually from 4.5% to 8.5%. However, in 2022, the isolation rate decreased slightly to 6.6%. At the same time, there was a significant increase in the resistance of *Acinetobacter* to ciprofloxacin from 18.9% to 75%, to gentamicin from 40% to 70.8%, to amikacin from 13.5% to 71%, and to meropenem and imipenem from 40% to 75%. A related work [52] reports the distribution of *Acinetobacter* by clinical department. Most isolates were recovered from the pediatric cardiac surgery department (67.8%), followed by the anesthesiology, resuscitation, and intensive care department (32.1%). Between 2020 and 2023, *Staphylococcus*, *Streptococcus*, *Enterococcus*, *E. coli*, *P. aeruginosa*, and *A. baumannii* were isolated from patients in the hematology department [53]. These studies confirm the major role of *E. coli* in urinary tract infections and the involvement of *Kl. pneumonia*, *P. aeruginosa*, *A. baumannii*, *E. coli*, *S. aureus*, pneumococci, and *Candida* spp. in the development of respiratory tract infections. *A. baumannii* was especially prevalent in cardiac surgery and intensive care units. Strains of *Kl. pneumonia*, *P. aeruginosa*, and *S. aureus* are more common in patients with surgical, therapeutic, and intensive care profiles, with *P. aeruginosa* being the most frequently detected. These pathogens have a high level of resistance to commonly used antibiotics.

In our study, we obtained 25 pure bacterial cultures from diverse biotopes, patient wounds and exudates, and medical equipment (Table 1). Viable cell counts ranged from 10^8^ to 10^10^ CFU/mL, and 85 percent of the strains were compatible, indicating suitability for further applications. Antibiotic susceptibility testing was performed against a panel of commonly used clinical antibiotics (Table 2). Multidrug resistance is of increasing concern due to the widespread use of antibiotics and the short time required for the development of resistance. Nearly 70% of bacterial pathogens are resistant to at least one antibiotic agent [54]. The strains we isolated exhibited resistance to between 2 and 14 antibiotics, ranging from 2 in *C. freundii* 9 BL, *P. aeruginosa* 13 BL, and *S. aureus* 20 BL to 14 in *Str. pneumoniae* 5 BL. These results were consistent with data from other studies [55].

The cultures we obtained may serve as candidates for the development of bacterial lysates and other applications, as they hold industrial value. They have an acceptable cell survival rate (above 10^6^ CFU/mL), exhibit multidrug resistance and mutual biocompatibility, grow well on both solid and liquid nutrient media, and are easily homogenized using a disintegrator. Worldwide, the ESKAPE pathogens are increasing the prevalence of hospital-acquired infections, which is additionally linked to high mortality rates among healthy individuals and patients with weakened immune systems [56]. Treatment options for these pathogens are limited due to the growing shortage of effective antibiotics, resulting from bacterial evolution manifested as multidrug resistance [57].

*Kl. pneumoniae* has emerged as a serious clinical and public health threat due to its increasing resistance to antimicrobial agents [8]. As an opportunistic pathogen, *Kl. pneumoniae* can cause a wide range of infections, including respiratory tract infections, urinary tract infections, and systemic infections. In healthcare settings, it is a leading cause of nosocomial infections [9]. *P. aeruginosa* [10] is found in hospitals, livestock farms, slaughterhouses, soil, aquatic environments, and wastewater. It is a major cause of hospital-acquired infections and associated mortality, particularly in immunocompromised patients and those with severe wounds, often leading to sepsis. *P. aeruginosa* is also a known cause of chronic lung infections in patients with cystic fibrosis or chronic obstructive pulmonary disease, catheter-associated urinary tract infections, and severe ventilator-associated pneumonia [58]. Bacteria of the genus *Acinetobacter* are among the most common pathogens responsible for severe healthcare-associated infections. Multidrug-resistant *A. baumannii* (MDR-AB) strains currently dominate worldwide [55], and their ability to rapidly acquire antimicrobial resistance poses significant treatment challenges [59,60]. Other clinically relevant species include *A. calcoaceticus*, *A. lwoffii*, *A. baylyi*, *A. haemolyticus*, *A. junii*, and *A. nosocomialis* [55].

Respiratory infections are a major factor in comorbidity in chronic obstructive pulmonary disease. Although significant improvements have been made in the treatment and control of the burden of these infections over the past few decades, there is still a shortage of vaccines against most infectious agents that cause respiratory tract infections; therefore, it is necessary to develop other preventive strategies [23]. One of the strategies for treating these infections is the use of BLs, which were introduced in the 1970s as oral vaccines for the prevention and treatment of respiratory infections. These lysates are mixtures of antigens derived from inactivated pathogens [28]. Lysates can affect the composition of the gut microbiota and indirectly exert immunomodulatory functions, providing an effective therapeutic option for patients with recurrent respiratory infections [55,61].

Lysates can be obtained using physical (mechanical), chemical, or enzymatic methods that disrupt cellular structures to release intracellular contents. After lysis, the process is the same for all methods: lyophilization (drying) and mixing in specified proportions. Enzymatic hydrolysis uses externally added enzymes (e.g., proteases, lyases) to break down bacterial cell walls and proteins, forming lysates [28,62]. This gentle method is mainly applied to lactic acid bacteria, bifidobacteria, and yeast, preserving free amino acids and vitamins while reducing toxicity. In contrast, autolysis relies on the cell’s own enzymes, activated under specific conditions, to induce self-destruction. Another method involves bacteriophages, which infect and lyse bacterial cells from within. Chemical methods include alkaline and acid lysis using alkalis and acids to destroy cell walls and membranes [63]. Chemical lysis allows precise control of lysis conditions (pH, temperature, reagent concentration). Physical methods provide rapid and complete destruction of cells, which leads to a high yield of lysate [28,64]. They include mechanical grinding with a mortar and pestle; ultrasonic treatment, which causes cavitation and cell wall disruption; freeze–thawing, where ice crystals damage membranes; microwave radiation, which heats and destroys cells; high-pressure homogenization through a narrow gap; and the Francis (ball mill) method, where cells are agitated with glass beads to break the walls.

The two most common methods for bacterial lysis are alkaline treatment and mechanical disruption. Alkaline lysis may denature bacterial antigen proteins, whereas mechanical methods preserve antigen structure in the lysate [28,30]. Suárez et al. provide a detailed comparison of these methods [28]. Each strain is cultured, inactivated using the selected procedure, lyophilized, and then mixed in fixed proportions to produce a polyvalent lysate. Alkaline lysis involves concentration, ultrafiltration, diafiltration, and drying. Cell wall disruption occurs through sodium hydroxide, which raises the pH to 11.5–12.5. Various NaOH concentrations have been tested in previous studies. Mechanical methods more commonly involve ultrasonic treatment or high-pressure homogenization. Earlier approaches included slow freezing, rapid thawing, and ultrasound. Finally, the lysis efficiency is verified by confirming the absence of viable cells (i.e., 100% destruction).

Based on our analysis of lysis methods, we selected a physical approach that is relatively easy to implement: homogenization using a French press, which generates high pressure. Compared to sonication or enzymatic lysis, the French press achieves over 90% cell disruption while preserving antigen integrity and avoiding chemical contamination [65]. This makes it an ideal method for preparing immunomodulatory lysates intended for therapeutic or prophylactic use.

The French press is user-friendly and does not require additional reagents, enzymes, or live cultures, thereby reducing the risk of lysate contamination. Consequently, it offers a safer alternative to chemical or enzymatic methods. While chemicals and enzymes used in lysis can remain in the final product and affect further studies or biological responses, this issue is avoided with the physical method. Chemical lysis, in particular, disrupts cell walls through harsh reagents, which may denature cellular structures and significantly reduce the immunogenicity of the lysate. A known drawback of physical methods, including the French press, is heat generation. Ultrasonic disintegration and homogenization can raise sample temperature, potentially leading to protein denaturation. To prevent this, a cooling system is used during the process. The operating principle of the French press involves a plunger that draws the bacterial suspension into the working cylinder of the disintegration head. The suspension is then forced through a narrow gap under high pressure, which can reach up to 1000 kg/cm^2^. The resulting shear forces and rapid pressure drop cause the rupture of cell walls and membranes.

The final consortium was free of contamination, maintained a viable cell count of 10^9^ CFU/mL, and showed a good yield. It was designed to target pathogenic microflora without disrupting the normal microbiota. The effectiveness of the developed bacterial lysate is based on the inclusion of clinically relevant strains that are major respiratory pathogens. These lysates could stimulate the immune system by promoting the recognition and clearance of pathogens. It is well established that bacterial lysates enhance host defense mechanisms by activating innate immunity, which helps reduce the frequency and severity of bacterial infections. A key indicator of their efficacy is the ability to activate macrophages and other immune cells, leading to a strengthened immune response. Thus, the physiological impact of such lysates is both targeted and beneficial.

The novelty of our lysate lies in the use of locally isolated clinical strains collected during the acute and postoperative phases of infection, the defined composition and ratio of strains within the consortium, and the implementation of a standardized high-pressure homogenization protocol. The use of a French press allows for efficient mechanical cell disruption while preserving antigenic integrity and avoiding chemical or enzymatic residues. A comparative analysis of the developed lysate and commercial products such as ismigen [28,66,67], imudon [68], and OM-85 [28,69,70] is presented in Table 6.

## 5. Conclusions

Antibiotic resistance is a global public health threat and a type of antimicrobial resistance. Addressing this issue requires coordinated efforts at the international level. The main antibiotic-resistant microorganisms include *S. aureus*, *Kl. pneumoniae*, *A. baumannii*, *P. aeruginosa*, *E. coli*, *Haem. influenzae*, *H. pylori*, and *Enterococcus* spp. The problem of antibiotic resistance has been confirmed by many studies conducted in various hospital departments, especially surgical units and intensive care units. The main preventive measures include controlling antibiotic use and developing new drugs, namely antibiotics and bacterial lysates, as alternative therapies.

In this study, 25 clinically significant, antibiotic-resistant bacterial strains were isolated from hospital patients and environments in Kazakhstan. These strains demonstrated high viability (up to 10^10^ CFU/mL), broad-spectrum multidrug resistance, and biocompatibility, which are essential characteristics for developing industrially valuable BLs. Among them, three priority pathogens (*Kl. pneumoniae* 12 BL, *P. aeruginosa* 3 BL, and *A. baumannii* 24 BL) were selected based on their clinical relevance, resistance profiles, and growth characteristics for inclusion in a polyvalent consortium.

A bacterial lysate was successfully obtained using a physical method of mechanical disruption using a high-pressure homogenizer (French press) under optimized conditions (three cycles, 100% power, with refrigeration). This approach ensured complete cell lysis while maintaining protein structure integrity and avoiding chemical contamination. Genotypic identification via 16S rRNA gene sequencing, complemented by biochemical tests, confirmed the taxonomic identity of the consortium strains.

The developed lysate is free from viable cells and contaminants and has potential applications in immunomodulatory therapy. It represents a localized and mechanically derived alternative to commercial BL products and supports future development of safe and effective biotherapeutics against respiratory and nosocomial infections caused by MDR pathogens. Future studies will focus on in vitro and in vivo analyses to validate its potential application.

## Figures and Tables

**Table 1:** Study objects, sources of microorganism isolation, and the maximum cell viability index.

Strains	Source Separation	Viability Index	
		×10^9^, CFU/mL	lg CFU/mL
*Escherichia coli* 1 BL	Wound washout	200 ± 0	11.3
*Staphylococcus haemolyticus* 2 BL	Oropharyngeal mucosa	16 ± 0.94	10.2
*Pseudomonas aeruginosa* 3 BL	Oropharyngeal mucosa	82 ± 0.94	10.91
*Staphylococcus haemolyticus* 4 BL	Urine	180 ± 9.4	11.25
*Streptococcus pneumoniae* 5 BL	Oropharyngeal mucosa	55.3 ± 0.27	10.74
*Escherichia coli* 6 BL	Urine	68 ± 0.94	10.83
*Staphylococcus epidermidis* 7 BL	Ear discharge swab	17.3 ± 0.27	10.23
*Citrobacter koseri* 8 BL	Urine	36 ± 0.94	10.56
*Citrobacter freundii* 9 BL	Oropharyngeal mucosa	30 ± 0.94	10.48
*Klebsiella pneumoniae* 10 BL	Oropharyngeal mucosa	11 ± 0.58	10.04
*Klebsiella pneumoniae* 11 BL	Oropharyngeal mucosa	14 ± 0.94	11.15
*Klebsiella pneumoniae* 12 BL	Purulent wound smear	54 ± 0.94	10.73
*Pseudomonas aeruginosa* 13 BL	Purulent wound smear	7 ± 1.41	9.84
*Pseudomonas aeruginosa* 14 BL	Tracheostomy tube flushing	19 ± 0.81	10.23
*Pseudomonas aeruginosa* 15 BL	Wound washout	36 ± 0.67	10.56
*Pseudomonas aeruginosa* 16 BL	Pleural cavity contents	96 ± 0.94	10.98
*Pseudomonas aeruginosa* 17 BL	Tracheostomy tube flushing	39 ± 4.24	10.55
*Staphylococcus aureus* 18 BL	Ear discharge swab	33.3 ± 0.38	10.48
*Staphylococcus aureus* 19 BL	Ear discharge swab	91 ± 0.58	10.96
*Staphylococcus aureus* 20 BL	Wound washout	9 ± 0.99	9.95
*Escherichia coli* 21 BL	Bile ducts	81 ± 0.47	10.91
*Escherichia coli* 22 BL	Wound washout	17 ± 0.47	10.23
*Escherichia coli* 23 BL	Urine	75 ± 0.47	10.87
*Acinetobacter* sp. 24 BL	Wound washout	76.6 ± 1.44	10.89
*Haemophilus influenzae* 25 BL	Pleural cavity contents	1	9.0

**Table 2:** Assessment of the resistance of the studied strains to antibacterial drugs from different groups (mm).

Strains	Inhibition Zone, mm													
	Mp	Ip	Axc	Amp	Bpc	Tc	Gm	Stm	Em	Lc	Cf	Ctr	Lf	Pf
*E. coli* 1 BL	11	12	0	0	0	6	7	10	0	15	16	0	10	0
*S. haemolyticus* 2 BL	18	23	0	6	0	0	9	14	8	16	16	0	14	6
*P. aeruginosa* 3 BL	16	15	0	0	0	0	14	7	0	0	15	0	7	0
*S. haemolyticus* 4 BL	0	0	0	6	0	6	23	18	0	7	21	0	0	0
*Str. pneumoniae* 5 BL	0	0	0	0	0	0	0	0	0	0	0	0	0	0
*E. coli* 6 BL	13	21	10	0	0	11	17	14	8	13	16	0	10	0
*S. epidermidis* 7 BL	15	0	0	0	0	16	14	8	0	8	16	0	14	6
*C. koseri* 8 BL	8	10	0	0	0	14	14	12	0	17	0	7	3	3
*C. freundii* 9 BL	18	17	11	8	0	7	7	16	0	0	8	7	6	10
*Kl. pneumoniae* 10 BL	18	0	0	0	0	7	20	16	0	21	12	0	2	3
*Kl. pneumoniae* 11 BL	17	19	0	0	0	15	18	16	0	22	9	9	16	0
*Kl. pneumoniae* 12 BL	0	0	0	0	0	0	15	15	0	17	17	17	16	0
*P. aeruginosa* 13 BL	9	9	9	0	11	6	18	21	20	11	13	10	15	0
*P. aeruginosa* 14 BL	16	15	0	0	11	0	6	0	20	18	7	20	21	0
*P. aeruginosa* 15 BL	15	18	0	0	7	15	25	14	13	20	12	8	0	0
*P. aeruginosa* 16 BL	10	0	0	8	0	7	17	7	9	0	6	10	16	0
*P. aeruginosa* 17 BL	7	16	0	0	0	13	12	9	10	15	7	10	16	0
*S. aureus* 18 BL	15	10	0	0	0	10	6	10	0	0	6	10	0	7
*S. aureus* 19 BL	14	16	0	0	0	0	7	10	0	16	7	10	6	10
*S. aureus* 20 BL	26	18	20	0	0	14	27	11	24	13	20	22	12	10
*E. coli* 21 BL	17	20	0	0	0	0	7	9	7	0	0	0	0	0
*E. coli* 22 BL	9	19	12	7	0	0	21	9	0	14	18	20	0	18
*E. coli* 23 BL	13	15	0	0	0	0	23	16	0	14	10	8	2	0
*Acinetobacter* sp. 24 BL	7	0	0	0	0	16	0	0	9	9	11	10	0	0
*Haem. influenzae* 25 BL	9	0	8	5	6	0	0	0	9	9	11	7	0	0

Note. Mp—meropenem; Ip—imipenem; Axc—amoxicillin; Amp—ampicillin; Bpc—benzylpenicillin; Tc—tetracycline; Gm—gentamicin; Stm—streptomycin; Em—erythromycin; Lc—chloramphenicol; Cf—cefepime; Ctr—ceftriaxone; Lf—levofloxacin; Pf—pefloxacin.

**Table 3:** Maximum cell survival rate after destruction.

Strains	Viability Index, CFU/mL		
	1st Cycle	2nd Cycle	3rd Cycle
*E. coli* 1 BL	10^8^	10^5^	10^2^
*S. haemolyticus* 2 BL	10^8^	10^5^	10^2^
*P. aeruginosa* 3 BL	10^7^	10^5^	10^3^
*S. haemolyticus* 4 BL	10^8^	10^5^	10^2^
*Str. pneumoniae* 5 BL	10^8^	10^5^	10^2^
*E. coli* 6 BL	10^7^	10^5^	10^3^
*S. epidermidis* 7 BL	10^7^	10^5^	10^2^
*C. koseri* 8 BL	10^8^	10^5^	10^2^
*C. freundii* 9 BL	10^8^	10^5^	10^2^
*Kl. pneumoniae* 10 BL	10^7^	10^5^	10^2^
*Kl. pneumoniae* 11 BL	10^8^	10^5^	10^2^
*Kl. pneumoniae* 12 BL	10^8^	10^5^	10^2^
*P. aeruginosa* 13 BL	10^7^	10^5^	10^2^
*P. aeruginosa* 14 BL	10^8^	10^5^	10^3^
*P. aeruginosa* 15 BL	10^8^	10^5^	10^2^
*P. aeruginosa* 16 BL	10^7^	10^5^	10^2^
*P. aeruginosa* 17 BL	10^8^	10^5^	10^2^
*S. aureus* 18 BL	10^8^	10^5^	10^2^
*S. aureus* 19 BL	10^7^	10^5^	10^3^
*S. aureus* 20 BL	10^8^	10^5^	10^2^
*E. coli* 21 BL	10^8^	10^5^	10^2^
*E. coli* 22 BL	10^7^	10^5^	10^2^
*E. coli* 23 BL	10^8^	10^5^	10^2^
*Acinetobacter* sp. 24 BL	10^8^	10^5^	10^2^
*Haem. influenzae* 25 BL	10^8^	10^5^	10^3^

**Table 5:** Biochemical analysis results of *Klebsiella* 12 BL.

Test	Glucose (Acid/Gas)	Lactose (Acid)	Dulcite (Acid)	Methyl Red	Voges- Proskauer	Citrate Utilization	Urease	Malonate
Result	+/+	+	−	−	+	+	+	+

**Table 4:** Bacterial lysate production modes.

No.	Mode (%Power), Number of Cycles	No.	Mode (%Power), Number of Cycles	No.	Mode (%Power), Number of Cycles
1	30% power, 1 cycle	6	100% power, 1 cycle	11	100% power, 2 cycles
2	55% power, 1 cycle	7	65% power, 2 cycles	12	80%–90%–100%
3	65% power, 1 cycle	8	75% power, 2 cycles	13	100%–90%–80%
4	75% 1 cycle	9	85% power, 2 cycles	14	90% power, 3 cycles
5	80% power, 1 cycle	10	90% power, 2 cycles	15	100% power, 3 cycles

**Table 6:** Comparison of the obtained BL.

Parameters	Ismigen [28,66,67]	Imudon [68]	OM-85 [28,69,70]	BL
Type of lysate	Mechanical	Chemical	Chemical	Mechanical
Indications	Treatment and prevention of diseases of the upper and lower respiratory tract	Treatment and prevention of diseases of the oral cavity and pharynx	Treatment and prevention of diseases of the upper and lower respiratory tract	Treatment and prevention of diseases of the upper and lower respiratory tract
Content	Lysates of 8 pathogenic bacteria	Lysates of 13 pathogenic bacteria	Lysates of 8 pathogenic bacteria	Lysates of 3 pathogenic bacteria

## Data Availability

The original contributions presented in the study are included in the article; further inquiries can be directed to the corresponding author.
